# Renal inhibition of miR-181a ameliorates 5-fluorouracil-induced mesangial cell apoptosis and nephrotoxicity

**DOI:** 10.1038/s41419-018-0677-8

**Published:** 2018-05-23

**Authors:** Xiao-Yun Liu, Fei-Ran Zhang, Jin-Yan Shang, Ying-Ying Liu, Xiao-Fei Lv, Jia-Ni Yuan, Ting-Ting Zhang, Kai Li, Xiao-Chun Lin, Xiu Liu, Qingqing Lei, Xiao-Dong Fu, Jia-Guo Zhou, Si-Jia Liang

**Affiliations:** 1 Department of Physiology, Key Laboratory of Cardiovascular Disease, School of Basic Medical Sciences, Guangzhou Medical University, Guangzhou, 511436 China; 2 Guangzhou Institute of Cardiovascular Disease, The Second Affiliated Hospital, Guangzhou Medical University, Guangzhou, 511436 China; 3 Department of Pharmacology, Cardiac and Cerebral Vascular Research Center, Zhongshan School of Medicine, Sun Yat-Sen University, Guangzhou, 510080 China; 4 Department of Cardiology, Sun Yat-Sen Memorial Hospital, Sun Yat-Sen University, Guangzhou, 510120 China; 5 Program of Kidney and Cardiovascular Disease, The Fifth Affiliated Hospital, Zhongshan School of Medicine, Sun Yat-Sen University, Guangzhou, 510080 China

## Abstract

The development of nephrotoxicity largely limits the clinical use of chemotherapy. MiRNAs are able to target various genes and involved in the regulation of diverse cellular processes, including cell apoptosis and death. Our study showed that miR-181a expression was significantly increased after 5-fluorouracil (5-FU) treatment in renal mesangial cells and kidney tissue, which was associated with decreased baculoviral inhibition of apoptosis protein repeat-containing 6 (BIRC6) expression and increased apoptotic rate. Enforced miR-181a expression enhanced 5-FU-induced p53-dependent mitochondrial apoptosis, including declined Bcl-2/Bax ratio, loss of mitochondrial membrane potential, cytochrome c release, and caspase-9 and caspase-3 activation. However, inhibition of miR-181a was associated with reduced p53-mediated mitochondrial apoptosis induced by 5-FU. Moreover, miR-181a increased BIRC6 downstream gene p53 protein expression and transcriptional activity by reducing ubiquitin-mediated protein degradation. We found that miR-181a directly targeted 3′-UTR of BIRC6 mRNA and negatively regulated BIRC6 expression. In vivo study, knockdown of miR-181a with adeno-associated virus harboring miR-181a-tough decoy attenuated 5-FU-induced renal cell apoptosis, inflammation and kidney injury. In conclusion, these results demonstrate that miR-181a increases p53 protein expression and transcriptional activity by targeting BIRC6 and promotes 5-FU-induced apoptosis in mesangial cells. Inhibition of miR-181a ameliorates 5-FU-induced nephrotoxicity, suggesting that miR-181a may be a novel therapeutic target for nephrotoxicity treatment during chemotherapy.

## Introduction

5-Fluorouracil (5-FU) is a potent antineoplastic agent widely used for the treatment of various malignancies because of its broad antitumor activity and synergistic action with other anticancer drugs^1^. However, unfortunately, 5-FU is designed to act via misincorporation of its metabolites into DNA and inhibition of thymine synthesis, and thus may not affect only cancer cells but also normal dividing cells of patients^1–3^. Consequently, it causes DNA damage, cell cycle termination, apoptosis and necrosis, and ultimately results in severe toxic effects and discontinuation of chemotherapy^4^. Of note, 5-FU gets catalyzed into dihydrouracil, which subsequently can be cleaved into α-fluoro-β-alanine, ammonia, urea, and carbon dioxide in liver, leading to nephrotoxicity^3,4^.

Mesangial cells play a critical role in maintaining the glomerular structural integrity and the function of the whole kidney, providing mesangial matrix homeostasis and regulating glomerular filtration^5^. It is reported that mesangial cell apoptosis can be observed during various chronic kidney diseases, including immunoglobulin A nephropathy, diabetic nephropathy, and lupus nephritis^5–7^. Moreover, the apoptosis of mesangial cells increases concomitantly with the severity of albuminuria and is directly involved in the pathogenesis of glomerulosclerosis^8^. These findings indicate that mesangial cell loss caused by apoptosis may contribute to the development of renal diseases. Importantly, mesangial cells are suggested to be susceptible to anticancer drugs such as 5-FU, and the apoptosis induced by 5-FU is considered to be associated with renal dysfunction^4,9^. However, the mechanisms of 5-FU-induced mesangial cell apoptosis are not fully understood.

Baculoviral inhibition of apoptosis protein repeat containing 6 (BIRC6), the largest member of the inhibition of apoptosis proteins (IAPs) family, contains a baculoviral IAP domain and a C-terminal ubiquitin-conjugating (UBC) enzyme domain^10^. BIRC6 like other IAPs promotes cell survival and inhibits apoptosis^11^. Another crucial regulator of cell apoptosis, p53, acts as a tumor suppressor by inducing cell apoptosis in most human tumors^12^. It has been reported that downregulation of p53 can ameliorate the cytotoxicity and nephrotoxicity induced by anticancer drugs^4,13^. Interestingly, p53 is a key downstream effector of BIRC6 and loss of BIRC6 triggers the upregulation of p53^11,14^. BIRC6 directly catalyzes p53 ubiqutiylation and proteasome degradation, which in turn inhibits mitochondria-dependent apoptosis^14^. These findings, together with the identification of BIRC6 as an upstream regulator of p53 raise the possibility that BIRC6 may be a novel target for the treatment of nephrotoxicity of 5-FU.

MicroRNAs (miRNAs) are small noncoding RNAs of 19–25 nucleotides in length that regulate gene expression by binding to the 3′-untranslated regions (3′-UTR) through mRNA translational repression or degradation^15^. Although previous studies showed that depletion of miRNAs by ablating Dicer, a key enzyme for miRNA maturation, results in a rapid progression to end-stage kidney disease^16–18^, only few miRNAs have been identified to be involved in renal dysfunction, such as 5-FU-induced nephrotoxicity^19,20^. In this study, we provided evidences that miR-181a regulates 5-FU-induced mesangial cell apoptosis through p53-dependent mitochondrial pathway by targeting BRIC6. Furthermore, we found that knockdown of miR-181a ameliorated 5-FU-induced nephrotoxicity.

## Materials and methods

### Materials and reagents

Fetal bovine serum (FBS), penicillin, streptomycin, lipofectamine 2000, TRIzol reagent, and 5,5′,6,6′-tetrachloro-1,1′,3,3′-tetraethyl-benza-midazolocarbocyanin iodide (JC-1) were obtained from Invitrogen (Carlsbad, CA, USA). RIPA lysis buffer, LDH leakage assay kit, BCA kit, enhanced chemiluminescence (ECL) kit, and antibodies against CD68 and Ly-6G were purchased from Beyotime Institute of Biotechnology (Shanghai, China). Unless otherwise indicated, all chemicals were purchased form Sigma-Aldrich (St. Louis, MO, USA).

### Cell culture

The immortalized human mesangial cell (HMC) line was kindly provided by Dr. Fengxian Huang (Sun Yat-Sen University)^21^. HCT116 p53^+/+^ or HCT116 p53^−/−^ cells were generous gifts from Dr. Ronggui Hu (Chinese Academy of Sciences). Cells were cultured in RPMI 1640 medium containing 10% FBS, 100 U/ml penicillin, and 100 μg/ml streptomycin. Cultures were maintained at 37 °C in a humidified incubator in a 95% O_2_ plus 5% CO_2_ atmosphere.

### miRNA transfection

miR-181a mimics, miRNA negative control, miR-181a inhibitor, or miRNA inhibitor negative control were obtained from Genepharma Biotech Company (Shanghai, China). The sequence for miRNA-181a mimics and inhibitor were as follows: miRNA-181a mimics, sense 5′-AACAUUCAACGCUGUCGGUGAGU-3′ and antisense 5′-UCACCGACAGCGUUGAAUGUUUU-3′; and miR-181a inhibitor, 5′-ACUCACCGACAGCGUUGAAUGUU-3′. miRNA negative control or miRNA inhibitor negative control shares no homologous region with the human genome sequences. All RNA duplexes were transfected into mesangial cells using lipofectamine 2000 according to the manufacturer's instructions.

### Luciferase reported assay

Based on the human BIRC6 mRNA sequence deposited in the GenBank database (accession no. NM_016252), the 3′-UTR of BIRC6 (1010 nt, 14,708–15,718) was amplified by PCR and subcloned in the psiCHECK-2 luciferase vector (Promega, Madison, WI, USA). The promoters of Bax containing the core promoter elements and p53 binding site were PCR amplified from genomic DNA and cloned in pGL3 enhancer reporter vector (Promega). Mesangial cells, HCT116 p53^+/+^ cells, or HCT116 p53^−/−^ cells were transfected with corresponding luciferase reporter and a control vector expressing β-gal using lipofectamine 2000. After 24 h, miR-181a mimics, miRNA negative control, miR-181a inhibitor, or miRNA inhibitor negative control was transfected into cells for 48 h. The cell lysates were assayed for luciferase activity using the Luciferase Assay System (Promega) with a luminescence counter (Centro SX3 LB 960, Berthold Technologies, Bad Wildbad, Germany). Luciferase activity was normalized by the corresponding β-gal luciferase activity.

### Western blotting

Western blotting was performed as previously described^22^. Briefly, mesangial cells were lysed with RIPA lysis buffer in the presence of protease inhibitor cocktail (Merck, Darmstadt, Germany). Cell debris were removed by centrifugation at 12,000 rpm for 12 min. The protein content was determined with BCA kit and separated by 8–10% SDS-PAGE, and then transferred to polyvinylidene fluoride (PVDF) membranes (Millipore, Bedford, MA, USA). The membranes were blocked in 5% (w/v) non-fat dry milk in Tris-buffered saline (TBS) and 0.1% (v/v) Tween 20 for 1 h at room temperature, and then incubated with the following primary antibodies at 4 °C overnight: BIRC6 (1:500) (Abcam, Cambridge, MA, USA); Ubiquitin, Bcl-2, Bax, cytochrome c, Cox IV, caspase-3 and caspase-9 (1:1000) (Cell Signaling Technology, Danvers, MA, USA); puma, Fas (1:500), p53, p21 and GAPDH (1:1000) (Santa Cruz Biotechnology, Paso Robles, CA, USA). After incubation of appropriate secondary horseradish peroxidase-conjugated antibodies including HRP-conjugated anti-rabbit or anti-mouse (1:1000; Cell Signaling Technology) for 1 h, blots were visualized with ECL kit and quantified with Image-Pro Plus 5.0 software.

### Quantitative real-time PCR

Total RNA was isolated from mesangial cells or kidney tissues using the TRIzol reagent according to the manufacturer’s instructions. An aliquot of 2 μg of RNA were reverse transcribed with a PrimeScript RT reagent kit (Bio-Rad Laboratories, Hercules, CA, USA), and real-time PCR was performed using SYBR Green PCR Master Mix (Bio-Rad Laboratories) on a MyiQ Single Color Real-time PCR Detection System (Bio-Rad Laboratories). The sequence-specific primers (Table S1) for the indicated genes were synthesized by Sangon Biothech (Shanghai, China). To detect miRNA expression, reverse transcription was performed using the TaqMan^®^ MicroRNA Reverse Transcription Kit (Applied Biosystems, Carlsbad, CA, USA) and PCR reactions were conducted using the TaqMan^®^ MicroRNA Assay Kit (Applied Biosystems). Primers for miR-181a, miR-181b, miR-181c, miR-181d, and the small nuclear RNA U6 were from RiboBio Co., Ltd. (Guangzhou, China). The fold change in expression for each gene was calculated using the 2^−ΔΔCT^ method, with 18S rRNA or U6 as an internal control. To examine the abundance of miR-181 family in mesangial cells and kidney tissues, the expression of miR-181a, miR-181b, miR-181c, miR-181d was assessed as absolute concentration based on a standard curve constructed with the use of synthetic miRNAs (GenePharma Biotech Company).

### Immunoprecipitation

Immunoprecipitation was performed as we previously described^23^. Cell lysates were immunoprecipitated with p53 antibody using protein A/G agarose (Santa Cruz Biotechnology). The immunoprecipitates were washed with lysis buffer three times and analyzed by western blotting with p53 or ubiquitin antibody.

### Cell viability assay

Cell viability was assessed using Cell Counting Assay Kit-8 (CCK-8, Dojindo Molecular Technologies, MD, Japan) as previously described^22^. Briefly, mesangial cells pretreated with or without miR-181a mimics, miRNA negative control, miR-181a inhibitor, or miRNA inhibitor negative control were subjected to 5-FU for 24 h. An aliquot of 10 μL of CCK-8 reagent was added to the medium for 2 h and the absorbance was read at 450 nm using a microplate reader (Bio-Tek, Winooski, VT, USA).

### Flow cytometry analysis

Apoptotic cells were quantified by the annexin V-allophycocyanin (APC)/7-amino-actinomycin D (7-AAD) double staining kit (KeyGEN Biotech, Nanjing, China) using flow cytometry. Briefly, cells were harvested, and were suspended in binding buffer containing APC-labeled annexin V and 7-AAD. After incubation in the dark at room temperature for 15 min, the samples were analyzed by a Beckman-Coulter EPICS XL-MCL flow cytometer (Beckman, Fullerton, CA, USA).

### Hoechst 33258 staining

The apoptotic morphology of mesangial cells was observed using Hoechst 33258 staining. Cells were fixed in 4% paraformaldehyde for 30 min and then were stained with Hoechst 33258 (20 μg/ml) for 5 min at room temperature in dark. Apoptotic cells were observed under a laser-scanning confocal microscopy (FV500, Olympus, Tokyo, Japan).

### Mitochondrial membrane potential detection

The mitochondrial membrane potentia (MMP) was measured using a membrane-permeant dye JC-1, which is a cationic dye that exhibits potential-dependent accumulation in mitochondria and emits red fluorescence (excitation, 550 nm; emission, 600 nm) in viable cells. In apoptotic cells, JC-1 outflows in cytoplasm as monomer, indicated by green fluorescence (excitation, 485 nm; emission, 535 nm). Consequently, the red/green fluorescence intensity ratio will decrease due to mitochondrial depolarization. Briefly, cells were incubated with JC-1 at 37 °C for 2 h in dark. After washing with PBS three times, the fluorescence was observed with a laser-scanning confocal microscopy (FV500, Olympus). The ratios of red/green fluorescence intensity were analyzed by Image-Pro Plus 5.0 software from at least six random microscopic fields of each sample.

### Mitochondria isolation

Intact mitochondria isolation was performed using Mitochondria Isolation Kit for Cultured Cells (Thermo Fisher Scientific Inc., Rockford, USA), according to the manufacturer’s protocol. Briefly, 800 μL of Reagent A was added to the cells and incubated for 2 min on ice. An aliquot of 10 μL of Reagent B was added to the each sample and incubated on ice for 5 min. During this period, samples were vortexed every minute. An aliquot of 800 μL of Reagent C was added followed by centrifugation at 700 × *g* for 10 min at 4 °C. The supernatant was transferred to a new tube and centrifuged at 12,000 × *g* for 15 min at 4 °C. Then the supernatant (cytosol fraction) were collected, while the pellet containing the isolated intact mitochondria was washed with 500 μL of Reagent C and centrifuged at 12,000 × *g* for 15 min at 4 °C. The cytosol and mitochondrial fractions were used for the western blotting. Cox IV was used as a loading control for the mitochondrial fraction.

### Adeno-associated virus (AAV) vectors

The miR-181a tough decoy (TuD) sequences (5′-ACTCACCGACAGCATCTGTTGAATGTT-3′) were synthesized and cloned into pDKD-CMV-eGFP-U6 plasmid by GenScript. The vector containing a upstream U6 promoter and a downstream 181a-TuD terminator was obtained by digesting with AgeI and EcoR1. This cassette was then packaged into capsids from AAV-9 serotype by transfection in HEK 293 cells to make AAV-TuD-181a. AAV-GFP (AAV serotype 9) vector harboring eGFP cDNA under the control of CMV promoter was obtained from SignaGen Laboratories (Rockville, MD, USA).

### Animal experiments

C57BL/6 mice (weighing ~20 g and aged 8–10 weeks) were purchased from Jackson Laboratories (Bar Harbor, ME, USA). All animal experiments was performed in accordance with the policies of the Sun Yat-Sen University Committee for Animal Research and conformed to the “Guide for the Care and Use of Laboratory Animals” of the National Institute of Health in China. For retrograde renal vein injection of AAV vectors, mice were anesthetized by intraperitoneal injection of pentobarbital sodium, and the left kidney was exposed through the abdominal incision between the point of the xiphoid cartilage and the navel. The renal vein was clamped off using a microvenous clip, and ~100 μL of AAV vector (1 × 10^11^ viral genome particles) expressing either TuD-181a or TuD-GFP was injected into the vein using a 33 G needle. Fifteen minutes later, the clamps were withdrawn and the renal vein was inspected for any evidence of leakage. The abdominal incision was sutured using a double layer of 6–0 silk sutures. After 14 days, the mice were received with intraperitoneal injection of 5-FU (200 mg/kg body weight) daily for 4 days. On the 18th day, mice were sacrificed, and the blood and kidney samples were collected for biochemical, immunohistochemical, and histopathological analysis.

### Assessment of blood parameters

Blood samples were collected and centrifuged for 10 min at 3000 × *g* to obtain serum. Serum blood urea nitrogen (BUN) and creatinine were measured spectrophotometrically by a fully automated clinical chemistry analyzer (Hitachi 7020, Tokyo, Japan) in Guangdong Medical Laboratory Animal Center. The LDH level in serum was determined using the LDH leakage assay kit according to the manufacturer’s instructions.

### Histologic and immunohistochemistry analyses

Kidney tissues were fixed in 10% formalin overnight and embedded in paraffin for sectioning at 5 μm thickness. Histology was performed on paraffin-embedded kidney tissues for hematoxylin & eosin (H&E) staining and periodic acid-schiff (PAS) staining. For immunohistochemical staining, kidney sections were incubated with primary antibodies overnight at 4 °C. Following 1 h incubation with biotinylated secondary anti-rabbit antibody at room temperature, sections were developed with 3,3-diaminobenzidine tetrachloride and counter-stained with hematoxylin. TUNEL staining was performed using ApopTag Apoptosis Kit (Millipore, Billerica, MA, USA) in accordance with manufacturer’s recommendations. All sections were subsequently examined with a light microscope (IX71, Olympus, Tokyo, Japan).

### Myeloperoxidase assay

Myeloperoxidase (MPO) activity of kidney tissues was determined as previously described^24^. Briefly, the tissues were homogenized with a tissue grinder and centrifuged at 15,000 × *g* for 15 min at 4 °C. The pellet was prepared in 50 mM KH_2_PO_4_ buffer (pH 6.0) containing 5 mM EDTA and 0.5% hexadecyl trimethyl ammonium bromide. Samples were centrifuged at 15,000 × *g* for 15 min at 4 °C. MPO activity of 10 μL of supernatant was measured, using 290 μL substrate solution (pH 6.0) containing *o*-dianisidine hydrochloride (0.167 mg/mL), 0.0005% hydrogen peroxide, and 50 mM KH_2_PO_4_ at 460 nm for 5 min, and was expressed as units per gram tissue.

### Statistical analysis

Results were expressed as mean ± SEM. Comparisons between groups were performed by an unpaired two-tailed Student’s *t* test or one-way ANOVA followed by Bonferroni multiple comparison post hoc test with a 95% confidence interval. *P* < 0.05 was considered to be statistically significant.

## Results

### Inhibition of miR-181a attenuates 5-FU-induced apoptosis in mesangial cells

**Fig. 1:**
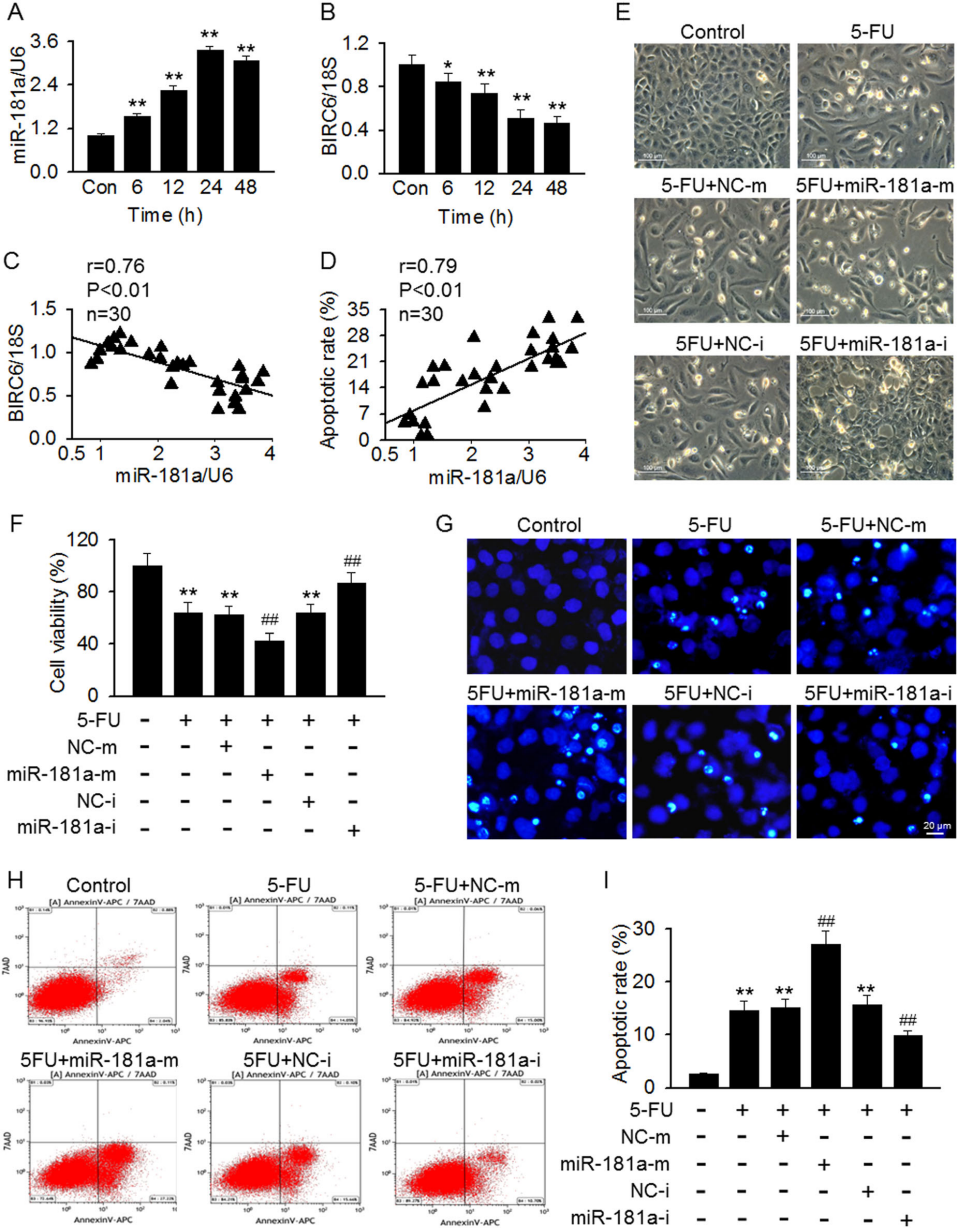
MiR-181a regulates 5-FU-induced mesangial cell apoptosis. **a**, **b** Analysis of miR-181a (**a**) and BIRC (**b**) in mesangial cells treated with 5-FU (100 μM) for 6, 12, 24, or 48 h. **p < 0.01 vs. control, *n* = 6. **c**, **d** miR-181a expression was inversely correlated with BIRC6 mRNA expression (**c**) and positively correlated with apoptotic rate (**d**). **e** The cells were pretreated with miR-181a mimics or miR-181a inhibitor for 48 h followed by incubation of 5-FU (100 μM) for another 24 h. Cell morphology was assessed by phase contrast microscopy. **f** Cell viability was assessed by CCK-8 assay. **p < 0.01 vs. control; ^##^*p* < 0.01 vs. 5-FU, *n* = 6. **g** Hoechst 33258 nuclei staining was used to detect apoptotic morphology. **h** Fluorescence-activated cell sorting-derived dot plot diagrams of mesangial cells stained with annexin V-APC/7-AAD. **i** Percentage of apoptotic cells was determined by quantitative analysis. **p < 0.01 vs. control;^##^*p* < 0.01 vs. 5-FU, *n* = 4

MiR-181a belongs to the miR-181 family, which comprises four major isoforms: miR-181a, miR-181b, miR-181c, and miR-181d. Quantitative real-time PCR (qRT-PCR) using absolute quantification showed that miR-181a is expressed highly both in mesangial cells and kidney tissues (Figure S1), indicating that miR-181a may play a critical role in regulating renal function. Interestingly, 5-FU treatment significantly increased miR-181a mRNA expression in mesangial cells. Exposure of 5-FU (100 μM) for 24 h resulted in a three-fold induction of miR-181a expression as compared with control group (Figure S2). As displayed in Fig. 1a, miR-181a expression was increased in a time-dependent manner when cells were incubated with 5-FU (100 μM) for up to 24 h. However, the protein expression of BIRC6 showed a contrary tendency (Fig. 1b). A significant correlation between miR-181a expression and BIRC6 mRNA expression or cell apoptotic rate was observed, respectively (Fig. 1c, d). To explore the role of miR-181a in 5-FU-induced apoptosis, we first observed the morphology of mesangial cells. The successful upregulation or inhibition of miR-181a in mesangial cells treated with miR-181a mimics or inhibitor was confirmed by qRT-PCR. No overexpression or downregulation of miR-181b, miR-181c, and miR-181d was observed (Figure S3). Compared with control group, 5-FU-treated cells showed a marked morphological changes including elongation of cells, large gaps in cell monolayer and increasing number of floating cells. These alterations were much more pronounced in miR-181a mimics-treated cells, but markedly alleviated after miR-181a inhibition (Fig. 1e). Further, CCK-8 assay revealed that miR-181a mimics transfection enhanced, whereas miR-181a inhibitor inhibited 5-FU that induced the decrease of cell viability (Fig. 1f). To verify these results, apoptotic bodies was visualized by using Hoechst 33258 staining. The formation of apoptotic bodies induced by 5-FU was dramatically augmented after miR-181a overexpression. However, downregulation of miR-181a markedly inhibited the formation of apoptotic bodies (Fig. 1g). Consistently, apoptotic rate after miR-181a mimics transfection was increased compared with 5-FU treatment group, while miR-181a inhibition led to the opposite results (Fig. 1h,i). These results indicate that the aggravation of 5-FU cytotoxicity by miR-181a in mesangial cells may be associated with reduced BIRC6 expression.

### MiR-181a activates p53-dependent mitochondrial apoptosis signaling

**Fig. 2:**
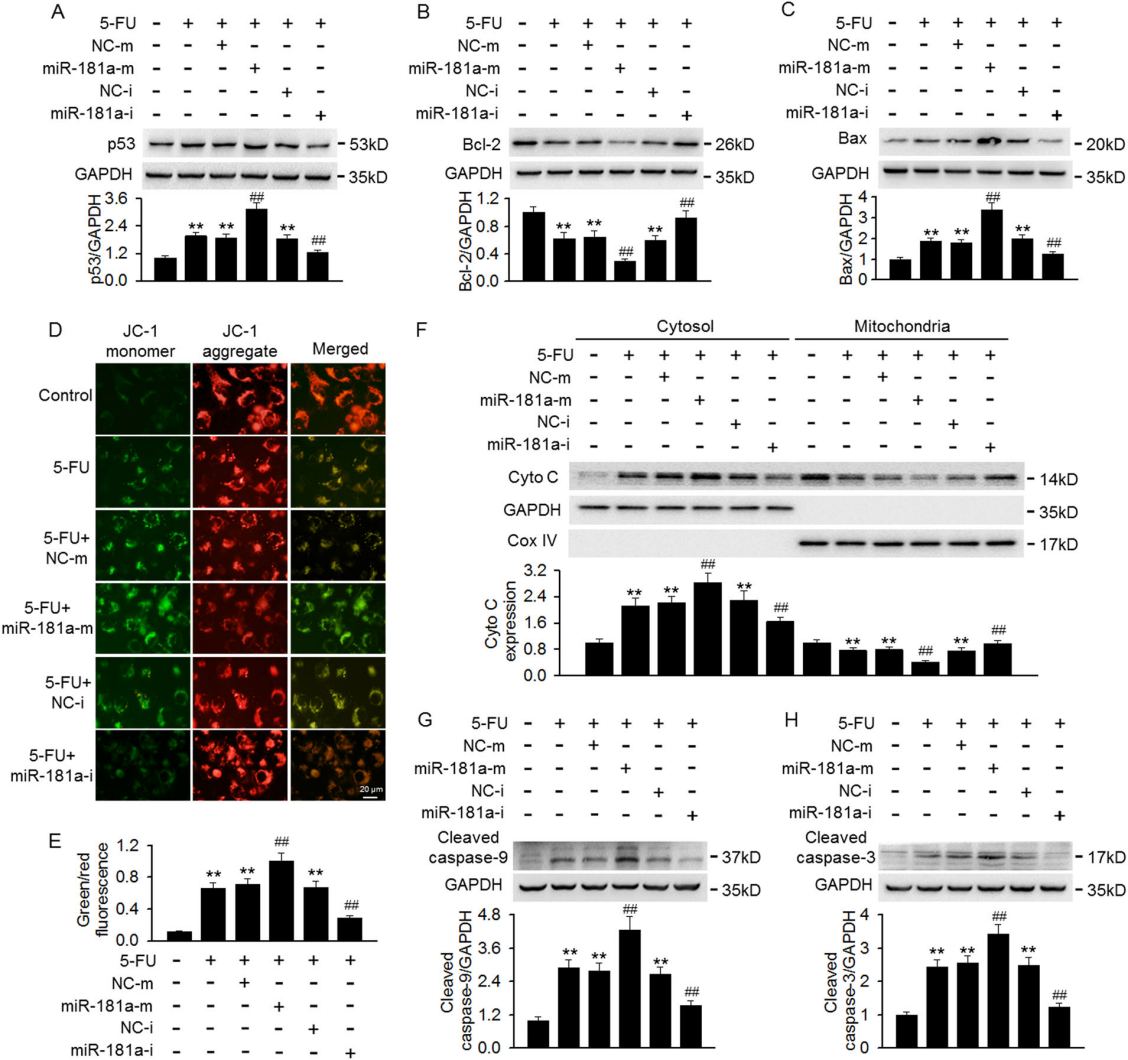
Effects of miR-181a on p53-dependent apoptosis signaling after 5-FU treatment. **a**–**c** Cells were pretreated with miR-181a mimics or miR-181a inhibitor for 48 h prior to incubation of 5-FU (100 μM) for another 24 h. The protein expression of p53 (**a**), Bcl-2 (**b**), and Bax (**c**) were determined by western blotting. **d** Mitochondrial membrane potential was measured by JC-1 staining. **e** Quantitative analysis of the green/red fluorescence intensity ratio, which represented as a surrogate marker of mitochondrial membrane depolarization. **f** Western blotting analyses of 5-FU-induced mitochondrial cytochrome c (Cyto c) release from mitochondria into the cytosol. **g**, **h** Caspase-9 (**g**) and caspase-3 (**h**) cleavage were detected by western blotting. **p < 0.01 vs. control; ^##^*p* < 0.01 vs. 5-FU, *n* = 6

Considering that p53, an important molecule in regulating cell apoptosis, we examined the effect of miR-181a on p53 and its related apoptosis proteins expression after 5-FU treatment. 5-FU strikingly increased the expression of p53 and its downstream pro-apoptotic signal protein Bax, whereas, the treatment decreased the expression of anti-apoptotic protein Bcl-2. MiR-181a overexpression further enhanced p53 and Bax expression and reduced Bcl-2 expression. On the contrary, inhibition of miR-181a attenuated p53 and Bax expression and restored Bcl-2 expression (Fig. 2a–c). Imbalance of Bcl-2/Bax ratio can cause changes in the inner mitochondrial membrane, leading to mitochondrial membrane depolarization^11,25^. To determine whether miR-181a-mediated aggravation of 5-FU cytotoxicity is associated with mitochondrial dysfunction, the effects of miR-181a on MMP were measured with JC-1 dye staining. The results of confocal microscopy showed that 5-FU markedly increased green fluorescence and decreased red fluorescence, leading to an increase of green/red fluorescence ratio, indicating a significant mitochondrial membrane depolarization. This depolarization was further enhanced after miR-181a overexpression, but counteracted by transfection of miR-181a inhibitor (Fig. 2d, e). Loss of MMP is known to induce pro-apoptotic cytochrome c release from mitochondria to cytoplasm^8^. Our results revealed that 5-FU treatment caused an obvious cytochrome c release from mitochondria to cytoplasm. MiR-181a overexpression augmented, whereas miR-181a inhibitor attenuated 5-FU that induced the release of cytochrome c (Fig. 2f). However, the total expression of cytochrome C remained unchanged across the different treatment conditions (Figure S4). Cytochrome c release from mitochondria can cleave caspase-9 and thus trigger caspase-3 activation, leading to mitochondria-dependent apoptosis^8,12^. Western blotting showed that 5-FU treatment significantly increased caspase-9 and caspae-3 cleavage, and this effect was further enhanced in cells overexpressed miR-181a. However, inverse results were observed in miR-181a inhibitor-treated cells (Fig. 2g, h). These data indicate that miR-181a promotes 5-FU-induced mesangial cell apoptosis through activation of p53 and its downstream mitochondria-dependent pathway.

### MiR-181a elevates p53 protein expression and transcriptional activity

**Fig. 3:**
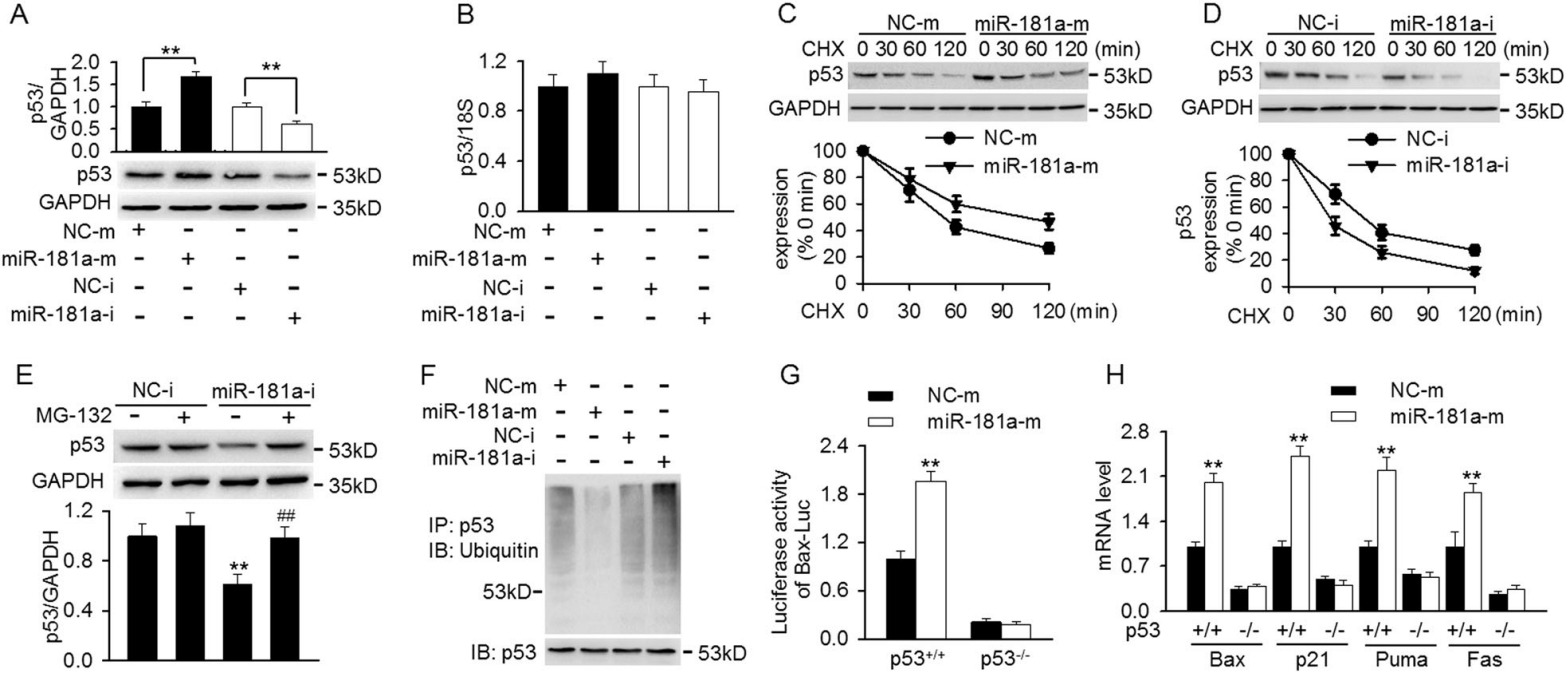
MiR-181a inhibition decreased p53 protein expression by facilitating ubiquitin-mediated degradation. **a**, **b** Cells were transfected with miR-181a mimics or miR-181a inhibitor for 48 h. The protein (**a**) and mRNA (**b**) expression of p53 were determined by western blotting and qRT-PCR, respectively. **p < 0.01 vs. miRNA negative control or miRNA inhibitor negative control, *n* = 5. **c**, **d** Mesangial cells pretreated with miR-181a mimics (**c**) or miR-181a inhibitor (**d**) were incubated with cycloheximide (CHX, 10 μg/ml) for indicated time period. Representative western blotting image of p53 and relative intensity of p53 protein expression were shown. **e** Cells were treated with or without MG-132 (20 μM) for 12 h prior to transfection with miRNA inhibitor negative control or miR-181a inhibitor for another 48 h. P53 protein expression was analyzed by western blotting. **p < 0.01 vs. miRNA inhibitor negative control; ^##^*p* < 0.01 vs. miR-181a inhibitor, *n* = 6. **f** Cell lysates were immunoprecipitated with p53 antibody and the ubiquitination of p53 was detected by western blotting using ubiquitin antibody. **g** HCT116 p53^+/+^ cells and HCT116 p53^−/−^ cells were co-transfected with miR-181a mimics or negative control and Bax luciferase (Bax-Luc) which contains the binding site of p53 for luciferase activity assay. **h** The mRNA expression of p53 target genes, including Bax, p21, Puma and Fas, was detected in HCT116 p53^+/+^ cells and HCT116 p53^−/−^ cells after miR-181a mimics transfection. **p < 0.01 vs. miRNA negative control, *n* = 6

We next investigated the mechanism by which miR-181a regulates p53-dependent mitochondrial apoptosis. As shown in Fig. 3a, transfection of miR-181a mimics increased, while miR-181a inhibitor decreased p53 protein expression, with a reciprocal pattern in BIRC6 expression. It is of note that neither miR-181a overexpression nor inhibition changed the mRNA expression of p53, suggesting that post-transcriptional regulation may not be involved (Fig. 3b). To further determined the mechanisms how miR-181a regulates p53 protein expression, mesangial cells were treated with cycloheximide (CHX, 10 μg/ml) for different times as indicated, and p53 protein stability was determined. Compared with miRNA negative control, overexpression of miR-181a dramatically decreased the rate of p53 degradation, resulting in prolonging p53 protein half-life more than 60 min (Fig. 3c). However, miR-181a inhibition significantly shortened the half-life of p53 protein, indicating miR-181a regulates the degradation of p53 rather than synthesis (Fig. 3d). Moreover importantly, in the presence of proteasome inhibitor MG-132, there was no significant difference between miRNA negative control and miR-181a inhibitor group, suggesting that miR-181a regulates p53 degradation via ubiquitin–proteasome pathway (Fig. 3e). Immunoprecipitation analysis showed that miR-181a overexpression markedly decreased the ubiquitination of p53, as detected by anti-ubiquitin antibody. On the contrary, p53 ubiquitination was increased in miR-181a inhibitor-treated cells (Fig. 3f). Since p53 is a transcription factor, we next examined whether miR-181a regulates p53 transcriptional activity. To this end, the luciferase activity of Bax containing the binding site of p53 was evaluated. Overexpression of miR-181a significantly increased the luciferase activities of the Bax-Luc reporter in HCT116 p53^+/+^ cells but not in HCT116 p53^−/−^ cells (Fig. 3g). Moreover, the mRNA level of Bax, p21, Puma and Fas, which are well-known p53 target genes, was increased after miR-181a mimics transfection, but remained unchanged in p53^−/−^ cells (Fig. 3h). Similarly, western blotting results also confirmed the induction of these p53-dependent genes by miR-181a mimics at protein level, indicating miR-181a increases p53 transcriptional activity (Figure S5A).

### MiR-181a directly targets BIRC6 and inhibits its expression

**Fig. 4:**
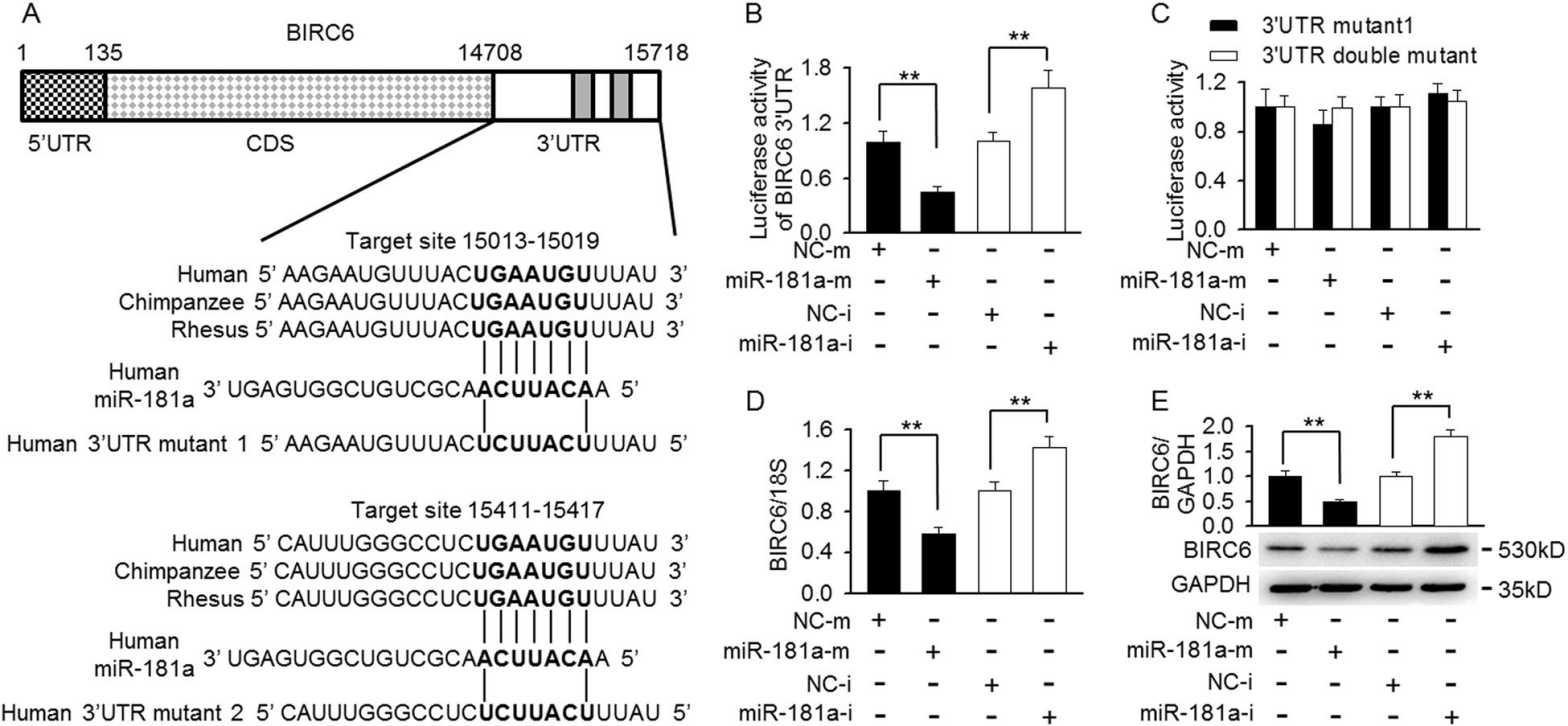
BIRC6 is regulated by miR-181a directly. **a** The schematic of the luciferase reporter containing the binding sites for miR-181a at the 3′-untranslated regions (3′-UTR) of BIRC6 from different species. The sequences of BIRC6 3′-UTR mutants used for luciferase reporter assay are presented. **b**, **c** Luciferase reporter constructs containing 3′-UTR (**b**) or mutant one 3′-UTR (3′-UTR mutant 1) or mutant double 3′-UTR (3′-UTR double mutant) (**c**) of BIRC6 was co-transfected with miRNA negative control (NC-m), miR-181a mimics (miR-181a-m), miRNA inhibitor negative control (NC-i), or miR-181a inhibitor (miR-181a-i) in mesangial cells and the luciferase activity were determined. **d**, **e** Mesangial cells were transfected with miR-181a mimics or miR-181a inhibitor for 48 h. qRT-PCR and western blotting were performed to examine endogenous BIRC6 mRNA (**d**) and protein (**e**) expression. **p < 0.01 vs. miRNA negative control or miRNA inhibitor negative control, *n* = 6

BIRC6, which serves as a p53 binding partner, has been documented to facilitate p53 degradation^14^. Indeed, co-immunoprecipitation assay showed that p53 interacted with BIRC6 (Figure S5B). Interestingly, using computational miRNA target prediction algorithms (TargetScan and Microcosm Targets), we found two miR-181a seed target regions within BIRC6 mRNA 3′-UTR (Fig. 4a). Luciferase reported assay showed that transfection of miR-181a mimics decreased the luciferase activity of BIRC 3′-UTR, whereas miR-181a inhibitor resulted in the opposite effect in mesangial cells (Fig. 4b). It was worthy to note that the luciferase reporter activity did not reach the level seen in the negative control when we initially only mutated the first miR-181a target site (15,013–15,019 nt). However, when the both miR-181a target sites were mutated, full luciferase activity was completely restored. Similarly, the increased luciferase reporter activity in cells-transfected miR-181a inhibitor was completely abolished when both target sites were mutated (Fig. 4c), suggesting that the second site (15,411–15,417 nt) may also binds to miR-181a. Consistent with BIRC6 3′-UTR reporter results, overexpression of miR-181a decreased, whereas inhibition of miR-181a increased the endogenous expression of BIRC6 at both mRNA and protein levels (Fig. 4d, e). Notably, the suppression of BIRC6 expression by miR-181 mimics was also observed in HCT116 p53^−/−^ cells (Figure S5C), indicating the inhibitory effect of miR-181a on BIRC6 expression is independent of p53.

### TuD knockdown of miR-181a prevents 5-FU-induced kidney injury

**Fig. 5:**
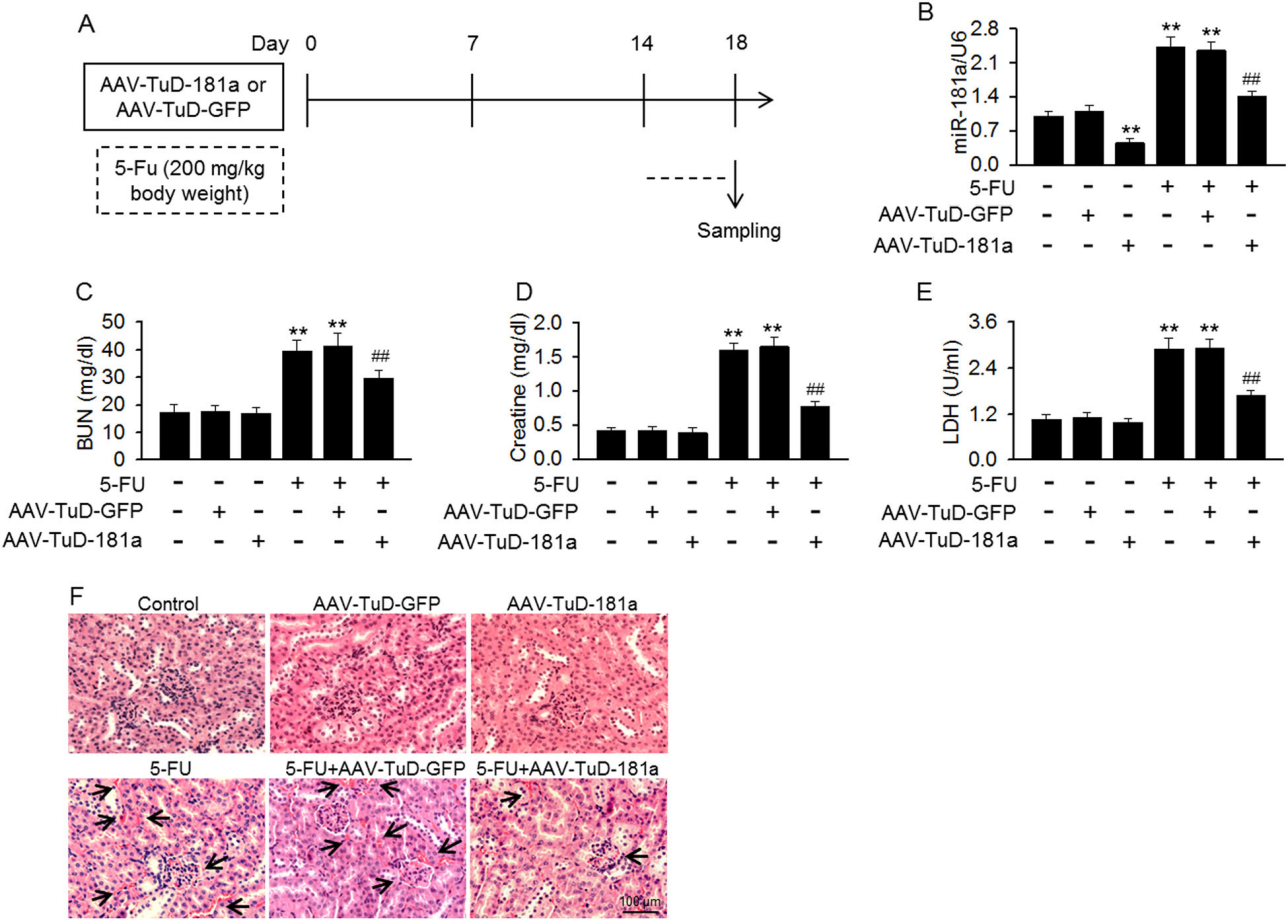
Knockdown of miR-181a alleviated kidney injury induced by 5-FU. **a** Experimental design to examine the effect of miR-181a inhibition on 5-FU-induced kidney injury. Mice were injected with adeno-associated virus encoding miR-181a tough decoy (AAV-TuD-181a) or AAV-TuD-GFP for 14 days and then treated with 5-FU (200 mg/kg body weight) for 4 days. **b** On the 18th day, mice were sacrificed, and the miR-181a expression in kidneys was determined. **c**–**e** Serum blood urea nitrogen (BUN) (**c**), creatinine (**d**), and LDH (**e**) concentration were tested by biochemical assay. **p < 0.01 vs. control; ^##^*p* < 0.01 vs. 5-FU, *n* = 5–10. **f** Histological examination of renal cortex section was performed using hematoxylin & eosin (H&E) staining. *n* = 5

The finding that miR-181a downregulation alleviated 5-FU-induced mesangial cell cytotoxicity in vitro promoted us to examine whether it played a similar role in vivo. Mice were injected with AAV expressing a TuD against miR-181a before induction of kidney injury by 5-FU (Fig. 5a). TuD-181a injection significantly decreased miR-181a expression in renal cortex and inhibited the increase of miR181a level induced by 5-FU, but did not alter the expression of miR-181b, miR-181c, and miR-181d (Fig. 5b and Figure S6). 5-FU administration resulted in an increase of serum BUN, creatinine and LDH concentration, which indicates the impairment of kidney function. However, they were decreased in 5-FU-treated mice injected with TuD-181a (Fig. 5c–e). Histological examination of renal cortex section with H&E staining showed that 5-FU significantly induced kidney injury, evidenced by blood sinusoids, interstitial hemorrhages as well as glomerular congestion, which was alleviated by TuD-181a injection (Fig. 5f). These results suggest that miR-181a downregulation attenuates 5-FU-induced nephrotoxicity.

### MiR-181a inhibition by TuD ameliorates renal inflammation induced by 5-FU

**Fig. 6:**
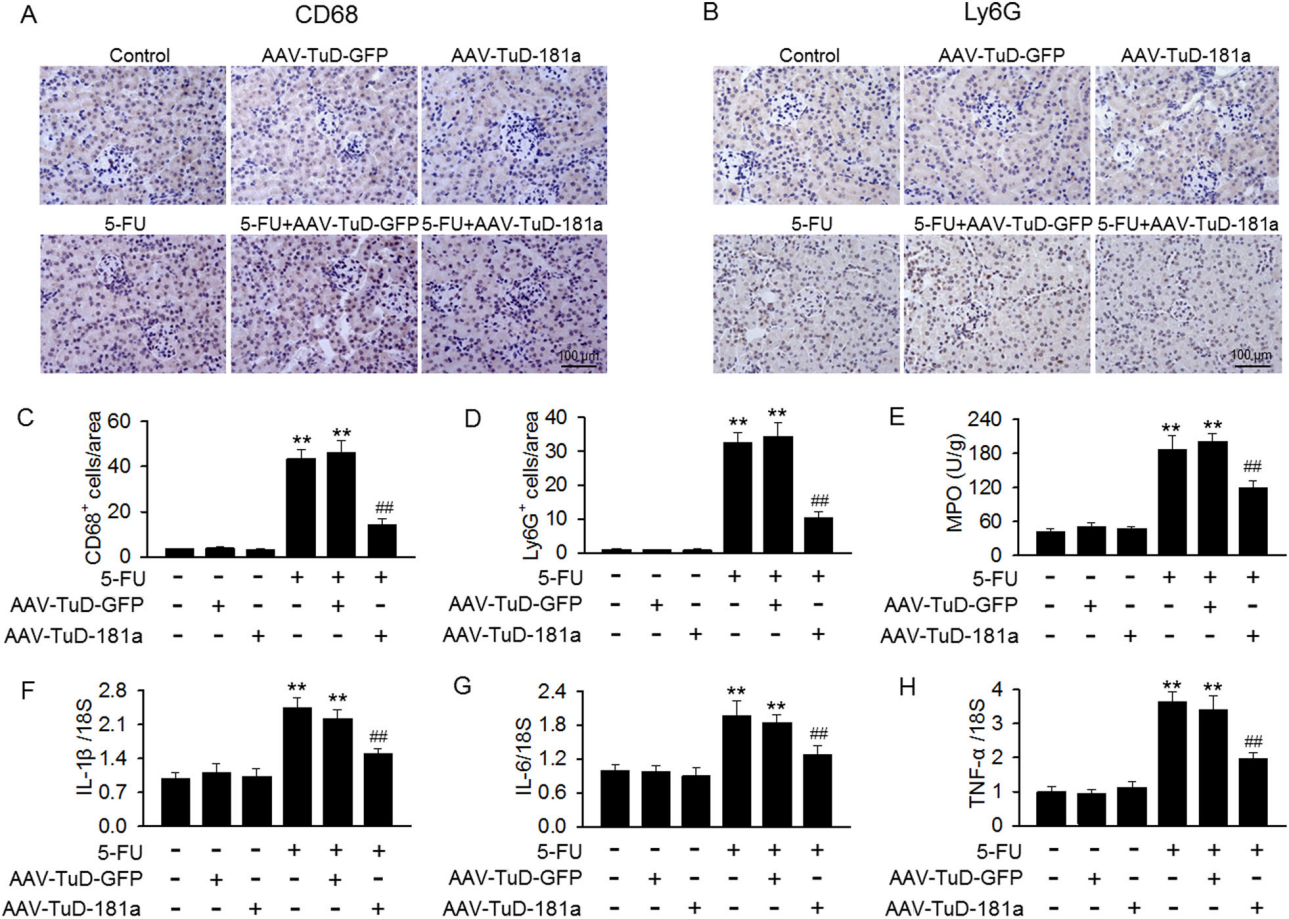
Renal inflammation induced by 5-FU was reduced by TuD-181a injection. **a**, **b** Immunohistochemical staining of Ly-6G (**a**) and CD68 (**b**) in kidney tissues of mice treated with TuD-GFP or TuD-181a under basal level or after 5-FU administration. **c**, **d** Quantification of Ly-6G-positive neutrophils (**c**) and CD68-positive macrophages (**d**) areas using Image-Pro 5.0 software. **e** The myeloperoxidase (MPO) activity in kidney tissues was determined. **f**–**h** The mRNA expressions of IL-1β (**f**), IL-6 (**g**), and TNF-α (**h**) were determined by qRT-PCR, respectively. **p < 0.01 vs. control; ^##^*p* < 0.01 vs. 5-FU, *n* = 4–8

Inflammation plays an essential role in kidney injury development. Indeed, the number of Ly-6G-positive neutrophils and CD68-positive macrophages was increased in renal cortex of mice after 5-FU treatment. Inhibition of miR-181a markedly decreased the infiltration of these cells into the kidney (Fig. 6a–d). MPO is an important enzyme which secreted by inflammatory cells including neutrophils and macrophages^3^. As expected, 5-FU treatment significantly increased the activity of MPO, and this increase was inhibited after knockdown of miR-181a (Fig. 6e). In addition, 5-FU induced the expression of pro-inflammatory cytokines, such as IL-1β, IL-6, and TNF-α, and was remarkably reduced in mice kidney after TuD-181a injection (Fig. 6f–h).

### Knockdown of miR-181a inhibits BIRC6/p53-dependent apoptosis pathway in 5-FU-induced nephrotoxicity

**Fig. 7:**
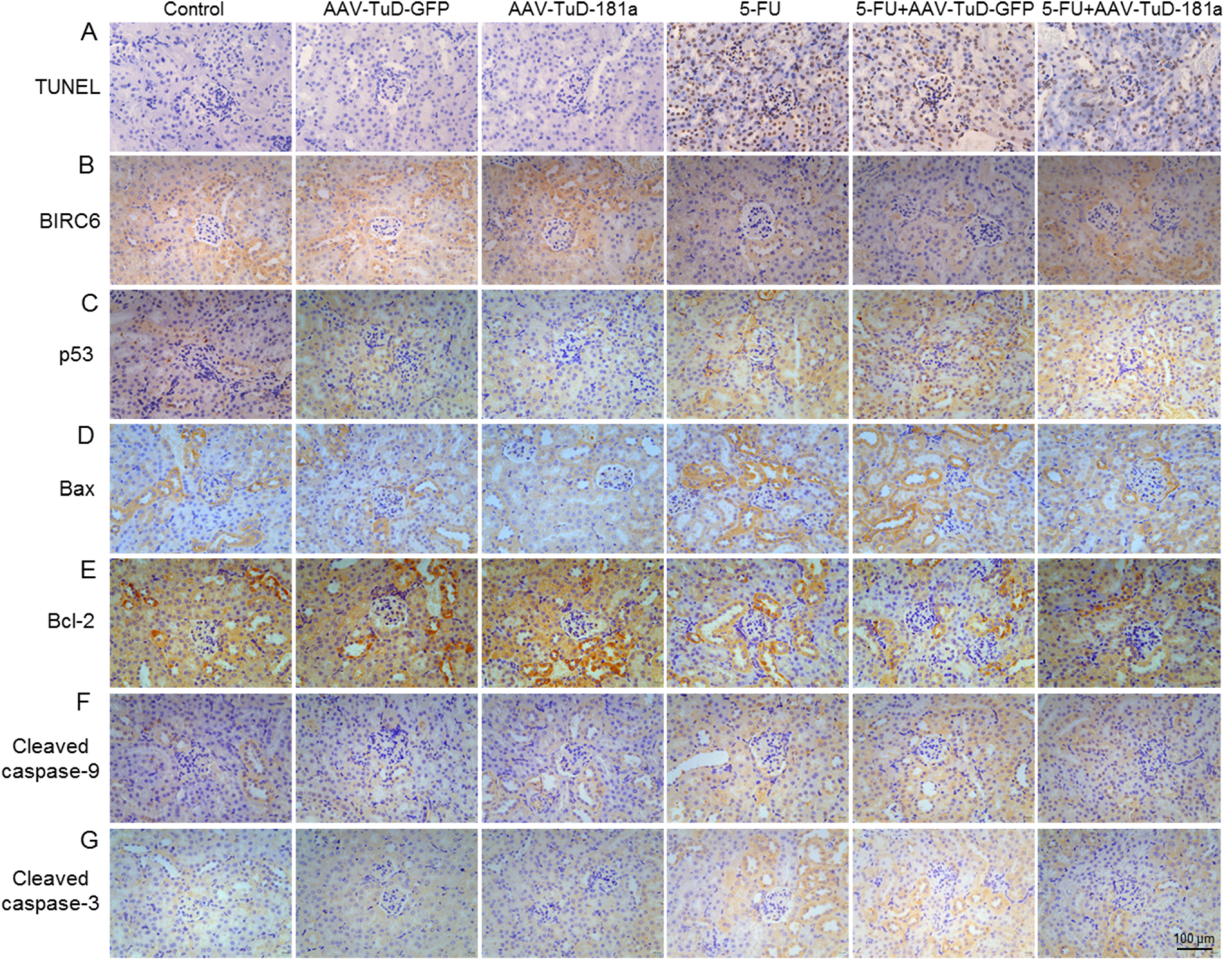
Effects of miR-181a inhibition on BIRC6/p53-dependent apoptosis in 5-FU-induced nephrotoxicity. **a**–**g** Histological analysis of renal cortex section from TuD-GFP- or TuD-181a-treated mice under basal level or after 5-FU administration, including TUNEL (**a**), BIRC6 (**b**), p53 (**c**), Bax (**d**), Bcl-2 (**e**), cleaved caspase-9 (**f**), and cleaved caspase-3 (**g**) staining. Representative images were shown from six independent experiments

To determine whether miR-181a inhibition attenuated 5-FU-induced apoptosis in kidney, renal apoptosis was examined using TUNEL assay. In accordance with the studies in vitro, TUNEL-positive apoptotic cells in renal cortex after 5-FU treatment were markedly decreased after TuD-181a treatment (Fig. 7a). We next evaluated the protein expression of key genes of the BIRC6/p53-dependent apoptosis pathway. As indicated by immunohistochemical staining, knockdown of miR-181a not only increased BIRC6 expression, but also inhibited 5-FU that induced the decrease of BIRC6 expression, further confirming the inhibitory effect of miR-181a on BIRC6 expression in vitro (Fig. 7b). Moreover, we also observed that the expression of p53 and pro-apoptotic genes such as Bax, cleaved caspase-9, and cleaved caspase-3 were increased in renal cortex of 5-FU-treated mice but anti-apoptotic Bcl-2 expression was decreased. However, the changes of these protein expression induced by 5-FU were significantly reversed in mice treated with TuD-181a (Fig. 7c–g).

## Discussion

In this study, we provided evidences demonstrating the critical function of miR-181a in regulating mesangial cell apoptosis and nephrotoxicity. Our results revealed that 5-FU treatment caused a marked upregulation of miR-181a expression, which was paralleled by decreased BIRC6 expression and increased apoptosis. MiR-181a is a novel miRNA that regulates p53 degradation, ubiquitination, and transcriptional activity by targeting BIRC6. Overexpression of miR-181a further enhanced 5-FU-induced apoptosis through p53-dependent mitochondrial pathway. Knockdown of miR-181a by TuD alleviated 5-FU-induced nephrotoxicity in mice.

Multiple anticancer drugs exert non-targeted action in dividing normal cells and ultimately leading to extensive side effects^2,4,19^. These clinical adverse effects such as cardiotoxicity, hepatotoxicity, and nephrotoxicity largely limit their wide therapeutic usage^3,26,27^. Thus, an understanding of the possible mechanisms of cellular toxicity is essential to amplify the therapeutic effectiveness in reducing the side effects induced by anticancer drugs. Interestingly, there is an accumulating body of evidence that miRNAs are involved in anticancer drug-induced kidney injury^15,19,20^. A longitudinal cohort study in malignant mesothelioma patients receiving over-time chemotherapy found that there was a marked increase in miR-21, miR-200c, and miR-423 expression in urinary, which suggests these miRNAs may be the biomarkers for acute kidney injury caused by anticancer drugs^28^. On the other hand, in a recent study involving miR-155-deficient mice, Pellegrini et al. reported a significantly higher level of kidney injury after cisplatin administration^29^. Moreover, miR-34a has been found to increase during cisplatin treatment in renal proximal tubular cells and this increase protects against renal cell apoptosis^19^. All these observations indicate that miRNAs have become new and important targets for the prevention of nephrotoxicity induced by chemotherapy.

The results of present study showed that miR-181a expression was obviously increased in mesangial cells and renal cortex after 5-FU treatment. MiR-181a expression was positively correlated with 5-FU-induced apoptosis. In addition, miR-181a overexpression enhanced 5-FU-induced apoptosis, while inhibition of miR-181a was associated with the reduced apoptotic rate, suggesting that the increased miR-181a expression may contribute to the apoptosis induced by 5-FU. This concurs with the discoveries that reduction of miR-181a could increase resistance to apoptosis in normal astrocytes and attenuate sensitivity of malignant glioma cells to radiation^25,30^. Here, we seek to increase resistance to apoptosis in mesangial cells, which is different with the desired effect in cancer cells. Moreover, the results of this study, in which we used in vivo mouse model, also showed that suppression of miR-181a protected renal cell against 5-FU cytotoxicity and nephrotoxicity. A large amount of evidence has accumulated that BUN, creatinine, and LDH are closely linked to the progression of kidney injury^4,13,19^. Our data revealed that 5-FU-induced kidney damage was accompanied by increased BUN, creatinine, and LDH level, which was in agreement with those reported previously^3,4^. However, lower level of BUN, creatinine, and LDH was observed in mice after TuD–miR-181a treatment. Further, inflammation has been shown to play an important role in kidney injury^3,6^. We found that knockdown of miR-181a was associated with a reduction of inflammation including inhibition of inflammatory cells infiltration, MPO activity, and a number of cytokine genes expression. Furthermore, we evidenced that miR-181a overexpression enhanced p53 expression and its downstream signaling Bax induced by 5-FU, and decreased Bcl-2 expression and MMP, concomitantly with increased cytochrome c release from mitochondria to cytoplasm and activation of caspase-9 and caspase-3. In contrast, the promotion effects of 5-FU on mitochondrial pathway of apoptosis was largely rescued by miR-181a inhibition. These results supported in vivo experiments, further demonstrating that miR-181a knockdown ameliorates 5-FU-induced renal cell cytotoxicity at least partially through inhibition of p53-dependent mitochondrial apoptosis pathway.

P53 is a short-lived protein with low abundance and transcriptional activity in unstressed cells. Upon genotoxic stress, p53 is upregulated by stabilization and accumulates in the nucleus to initiate mitochondrial pathway and promote apoptosis^12^. BIRC6 is an important upstream regulator of p53, which has been reported by embryonic lethality induced by BIRC ablation that can be rescued by p53 downregulation^11^. Deletion of C-terminal half of BIRC6 causes p53 stabilization and leads to apoptosis^11^. Recent study in hepatoma cells showed that BIRC6 could act similarly to Mdm2, Pirh2, and COP1, directly catalyzing p53 ubiquitination and proteasome degradation^14^. These studies suggest that the association between BIRC6 and p53 auto-regulatory feedback loop is critical for p53-dependent activation of the mitochondrial pathway. Indeed, previous studies have reported that BIRC6 silence induces apoptosis in cancer cells and mouse embryonic fibroblasts through upregulation of p53 and activation of mitochondria-dependent apoptosis pathway^11,14^. MiRNAs has been well recognized to involve in the regulation of multiple cellular processes including cell apoptosis and death^19,25,28,30–32^. To date, a few miRNAs such as miR-342 and microRNA BART15-3p have been identified to function by directly targeting BIRC6 and thus regulate cell apoptosis^31,32^, indicating these miRNAs are important mediators for BIRC6 that can become an additional mechanism for the regulation of p53-dependent mitochondrial apoptosis.

In the current study, for the first time, we found that BIRC6 is a novel target of miR-181a, as identified by luciferase reporter assay. Overexpression of miR-181a decreased BIRC6 expression by binding to its 3′-UTR, whereas inhibition of miR-181a led to the opposite results. Correspondingly, our study showed that upregulation of miR-181a increased p53 protein expression, which exhibited a reciprocal pattern of BIRC6 expression. However, qRT-PCR analysis showed that neither miR-181a mimics nor inhibition changed p53 mRNA expression, indicating that the transcriptional regulation may not take place. Given the important role of BIRC6 in regulating p53 ubiquitination and proteasome degradation^14^, it would be interesting to determine whether miR-181a increases p53 protein expression by protein stabilization. Our data found that inhibition of miR-181a shortened, whereas overexpression of miR-181a extended the half-life of p53 protein. Moreover, we further verified that miR-181a downregulation promoted p53 degradation via ubiquitin–proteasome pathway, because the decrease in p53 protein expression induced by miR-181a inhibition was abrogated completely by proteasome inhibitor MG-132 and p53 ubiquitination was dramatically increased after miR-181a downregulation. These results suggest that miR-181a negatively regulates BIRC6, which in turn inhibits p53 degradation and increases its protein expression and transcriptional activity. Interestingly, it is noted that miR-181a also displayed a p53-independent signal on apoptosis. Recent study revealed that miR-181a targets Bcl-2 family members, Bcl-2, Bim, and Mcl-1 in astrocytes, leading to mitochondrial dysfunction and apoptosis^25,30^. In addition to BIRC6 and p53, the regulation of Bcl-2 family members and other unidentified targets may contribute together to miR-181a-mediated apoptosis by a p53-independent pathway. The crosstalk between these targets and p53 should be further investigated to unveil the critical role of miR-181a in apoptosis.

In summary, our study demonstrates that miR-181a promotes 5-FU-induced mesangial cell apoptosis through p53-dependnet mitochondrial apoptosis by targeting BIRC6. These results suggest that inhibition of miR-181a may be a potential strategy to prevent 5-FU-induced nephrotoxicity.

## Electronic supplementary material

Supplemental Materials
