# Supplementary material for: Renal inhibition of miR-181a ameliorates 5-fluorouracil-induced mesangial cell apoptosis and nephrotoxicity
Source: Cell Death Dis. 2018 May 23;9(6):610. doi: 10.1038/s41419-018-0677-8 (PMC5966400; doi:10.1038/s41419-018-0677-8)
Supplement: Supplementary file 1 — Supplemental Materials [file 41419_2018_677_MOESM1_ESM.doc]

**Renal inhibition of miR-181a ameliorates 5-fluorouracil-induced mesangial cell apoptosis and nephrotoxicity**

Xiao-Yun Liu*^1,2^, Fei-Ran Zhang*^3^, Jin-Yan Shang*^3^, Ying-Ying Liu^3^, Xiao-Fei Lv^3^, Jia-Ni Yuan^3^, Ting-Ting Zhang^3^, Kai Li^3^, Xiao-Chun Lin^3^, Xiu Liu^3^, Qingqing Lei^3^, Xiao-Dong Fu^1,2^, Jia-Guo Zhou^1,2,3,4,5^, Si-Jia Liang^3^

^1^Department of Physiology, Key Laboratory of Cardiovascular disease, School of Basic Medical Sciences, Guangzhou Medical University. ^2^Guangzhou Institute of Cardiovascular Disease, The Second Affiliated Hospital, Guangzhou Medical University, 511436, China. ^3^Department of Pharmacology, Cardiac and Cerebral Vascular Research Center, Zhongshan School of Medicine, Sun Yat-Sen University. ^4^Department of Cardiology, Sun Yat-sen Memorial Hospital, Sun Yat-Sen University. ^5^Program of Kidney and Cardiovascular Disease, The Fifth Affiliated Hospital, Zhongshan School of Medicine, Sun Yat-Sen University, Guangzhou, 510080, China.

Running title: MicroRNA-181a and nephrotoxicity

Please address inquiries to:

Si-Jia Liang or Jia-Guo Zhou, Department of Pharmacology, Cardiac and Cerebral Vascular Research Center, Sun Yat-Sen University, 74 Zhongshan 2 Rd, Guangzhou, 510080 or Department of Physiology, Key Laboratory of Cardiovascular disease, School of Basic Medical Sciences, Guangzhou Medical University, Xinzao, Panyu District, Guangzhou, 511436, China.

Tel: 86-20-87331857, Fax: 86-20-87331209.

E-mail: [liangsj5@mail.sysu.edu.cn](mailto:liangsj5@mail.sysu.edu.cn) or zhoujg@ mail.sysu.edu.cn

*Liu XY, Zhang FR and Shang JY contributed equally to this work

**Supplemental files**

**Table S1. Primers used for qRT-PCR**

| Genes | primers | sequences |
| --- | --- | --- |
| *BIRC6* | Forward  Reverse | 5’-GACTTCACTTCCGGCTAACG-3’  5’-CGCTCAGCACAATCACACTG-3’ |
| *p53* | Forward  Reverse | 5’-GAACAAGTTGGCCTGCACTG-3’  5’-GAAGTGGGCCCCTACCTAGA-3’ |
| *Bax* | Forward  Reverse | 5’-AGAACCATCATGGGCTGGAC-3’  5’-CAGTCGCTTCAGTGACTCGG-3’ |
| *p21* | Forward  Reverse | 5’-CCGTCTCAGTGTTGAGCCTT-3’  5’-CCTGGAGCTGAGAGGGTACT-3’ |
| *puma* | Forward  Reverse | 5’-TGGGTGAGACCCAGTAAGGA-3’  5’-TAAGGGCAGGAGTCCCATGA-3’ |
| *Fas* | Forward  Reverse | 5’-CCTGCCAAGAAGGGAAGGAG-3’  5’-AAGACAAAGCCACCCCAAGT-3’ |
| *IL-β* | Forward  Reverse | 5’-TGCCACCTTTTGACAGTGATG-3’  5’-GGAGCCTGTAGTGCAGTTGT-3’ |
| *IL-6* | Forward  Reverse | 5’-GATGCTGGTGACAACCACGG-3’  5’-TCTGTGACTCCAGCTTATCTGTTA-3’ |
| *TNF-α* | Forward  Reverse | 5’-AGGGGATTATGGCTCAGGGT-3’  5’-TGCACCTCAGGGAAGAATCTG-3’ |
| *18S rRNA (human)* | Forward  Reverse | 5'-CGGCTACCACATCCAAGGAA-3'  5'-CTGGAATTACCGCGGCT-3' |
| *18S rRNA (mouse)* | Forward  Reverse | 5'-GCAATTATTCC CCATGAACG-3'  5'-GGCCTCACTAAACCATCCAA-3' |

**Supplementary Figures**

**Figure S1**

**Figure S1. MiR-181a is the dominantly expressed in miR-181 family in mouse kidney.** (A and B) MiR-181a, miR-181b, miR-181c and miR-181d in mesangial cells (A) and mouse kidney tissues (B) was determined using qRT-PCR based absolute quantification methods.

**Figure S2**

**Figure S2. 5-FU induced miR-181a expression in mesangial cells.** The cells were treated with various concentrations of 5-FU (50, 100, 200 or 400 μM) for 24 h, miR-181a expression was examined by qRT-PCR. **p<0.01 vs. control, n=6.

**Figure S3**

**Figure S3. Effects of miR-181a upregualtion or inhibition on miR-181 family expression.** (A and B) Mesangial cells were transfected with miR-181a mimics (A) or miR-181a inhibitor (B) for 48 h. QRT-PCR analysis of miR-181a, miR-181b, miR-181c and miR-181d mRNA expression. **p<0.01 vs. corresponding negative control, n=6.

**Figure S4**

**Figure S4. Effects of miR-181a on the total expression of cytochrome C after 5-FU treatment.** Cells were pretreated with miR-181a mimics or miR-181a inhibitor for 48 h prior to incubation of 5-FU (100 μM) for another 24 h. The total protein expression of cytochrome c was detected by western blotting. n=6.

**Figure S5**

**Figure S5. MiR-181a increased p53 transcriptional activity and decreased BIRC6 expression independently of p53.** (A) HCT116 p53^+/+^ or HCT116 p53^-/-^ cells were transfected with miR-181a mimics or miRNA negative control for 48 h. The protein expression of Bax, p21, Puma, Fas and p53 were determined by western blotting. (B) Western blotting (IB) analysis for BIRC6 after immunoprecipitation (IP) with p53 antibody in mesangial cells lysates. (C) HCT116 p53^+/+^ or HCT116 p53^-/-^ cells were transfected with miR-181a mimics or miRNA negative control for 48 h. Western blotting analysis of BIRC6 and p53 expression. **p<0.01 vs. corresponding negative control, n=4.

**Figure S6**

**Figure S6. Inhibition of miR-181a by tough decoy (TuD) had no effect on miR-181b, miR-181c and miR-181d expression.** Mice were injected with adeno-associated virus encoding miR-181a TuD (AAV-TuD-181a) or AAV-TuD-GFP for 18 days. Expression of miR-181b, miR-181c and miR-181d was determined by qRT-PCR.
